# Total knee arthroplasty with corrective osteotomy for knee osteoarthritis associated with malunion after tibial plateau fracture: a case report

**DOI:** 10.1186/s13104-017-2553-5

**Published:** 2017-06-26

**Authors:** Toshihiro Hosokawa, Yuji Arai, Shuji Nakagawa, Toshikazu Kubo

**Affiliations:** Department of Orthopaedics, Graduate School of Medical Science, Kyoto Prefectural University of Medicine, Kawaramachi-Hirokoji, Kamigyo-ku, Kyoto, 602-8566 Japan

**Keywords:** Total knee arthroplasty, Corrective osteotomy, Osteoarthritis

## Abstract

**Background:**

When surgeons perform total knee arthroplasty in patients with knee osteoarthritis due to malunion following fractures around the knee joint, corrective osteotomy is recommended for severe deformities. Most such deformities are coronal plane varus or valgus deformities, and reports of sagittal plane flexion or extension deformities are rare. We describe a case in which a one-stage total knee arthroplasty was performed with extension corrective osteotomy in the sagittal plane.

**Case presentation:**

A 71-year-old Japanese man presented with left knee pain. He had knee osteoarthritis associated with malunion after a tibial plateau fracture. Plain radiography showed a varus deformity in the coronal plane and a marked flexion deformity in the sagittal plane. We performed total knee arthroplasty concurrently with extension corrective osteotomy using a long stem. Full weight bearing was permitted at 6 weeks postoperatively, and the patient was able to walk without assistance.

**Conclusions:**

This surgical method appears to be beneficial for shortening the duration of treatment and improving knee function.

## Background

When surgeons perform total knee arthroplasty (TKA) in patients with knee osteoarthritis (OA) due to malunion following fracture around the knee joint, corrective osteotomy is recommended for severe deformities [1, 2]. Most such deformities are coronal plane varus or valgus deformities, and reports of sagittal plane flexion or extension deformities are extremely rare. Here, we present the case of a patient with knee OA associated with malunion after a tibial plateau fracture in which we performed a one-stage TKA with extension corrective osteotomy.

## Case presentation

**Fig. 1:**
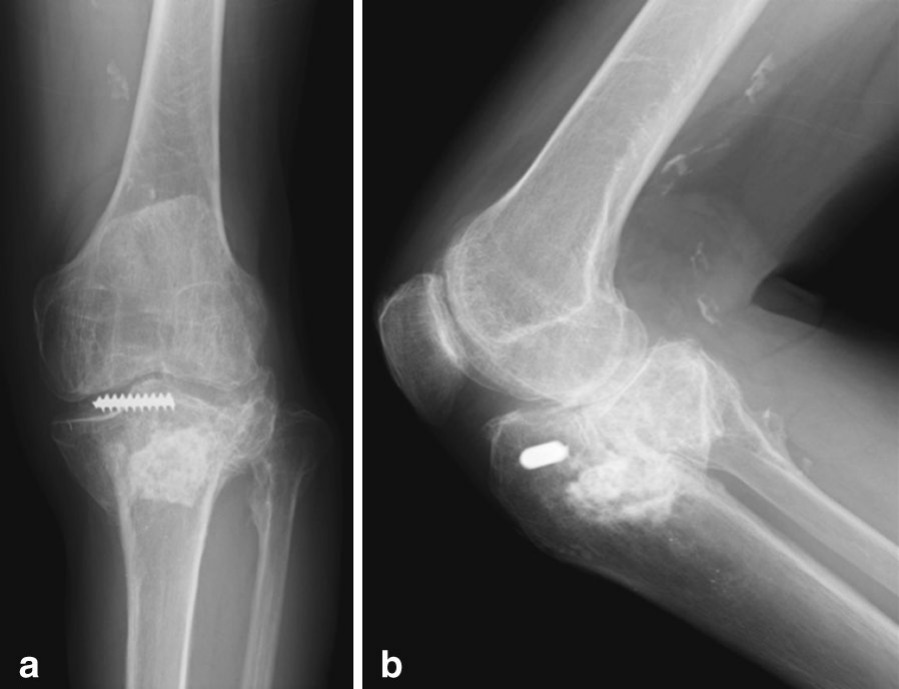
Radiographs of the left knee. **a** Anteroposterior view shows a shortened proximal end of the tibia due to the fracture and varus deformity. **b** Lateral view shows a collapsed articular surface of the posterior tibia with marked posterior angulation

The patient was a 71-year-old Japanese man who, 21 years earlier, had fractured the left tibial plateau and underwent open osteosynthesis. He did not experience symptoms until 21 years after the injury, when he became aware of pain in the left knee joint and visited our clinic for the first time. Range of motion in the left knee joint was −5° extension to 135° flexion. An anteroposterior radiograph of the left knee showed degenerative joint changes. The tibia had shortened due to the fracture. A lateral radiograph showed that the posterior articular surface of the tibia had collapsed and had significant posterior angulation. Deformities were identified as a 9° of varus deformity and a 27° of flexion deformity (Fig. 1).

**Fig. 2:**
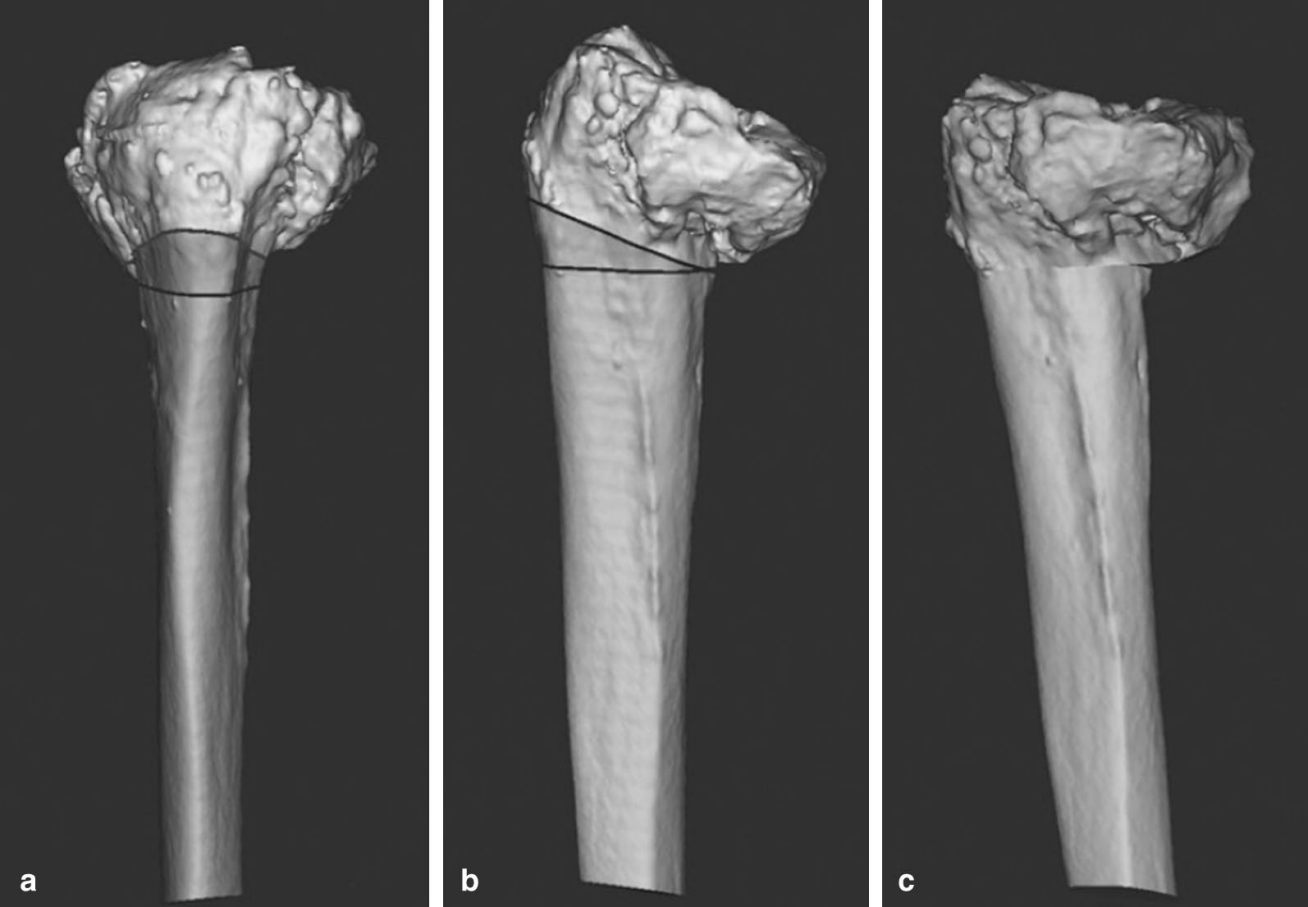
Simulation of corrective osteotomy with three-dimensional image-processing software. **a** Anteroposterior view. **b** Lateral view. **c** Improved posterior angulation after simulation of corrective osteotomy

**Fig. 3:**
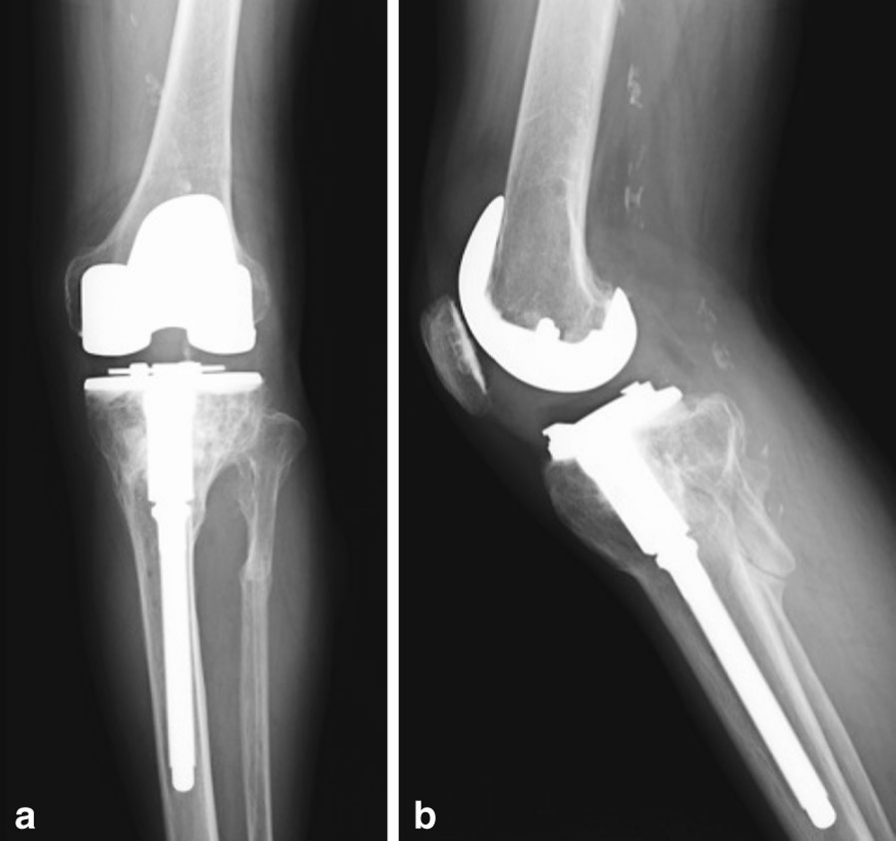
Radiographs at 24 months postoperatively. **a** Anteroposterior view. **b** Lateral view

If tibial osteotomy was performed perpendicular to the tibial axis using the lowest point of the tibial articular surface as a reference point, there was a risk of cutting the tibial tuberosity. Conversely, tibial osteotomy performed proximal to the lowest point of the tibial articular surface would markedly decrease the articular surface coverage by the implant. For these reasons, we decided that extension corrective osteotomy was necessary. We performed a preoperative simulation using a three-dimensional (3D) model with 3D image-processing software. The line that passes through the hinge point perpendicular to the tibial bone axis was identified as the osteotomy line, and we planned to perform a 13° corrective closing osteotomy. The width of the anterior tibia necessary for corrective osteotomy was 10 mm (Fig. 2). We created a life-size 3D bone model and performed a mock surgery. The medial parapatellar approach was used as the method for open surgery. We dissociated soft tissue from the deep layer of the medial collateral ligament to the enthesis of the semimembranosus muscle, adjusted the balance of medial and lateral soft tissue, and subsequently performed a distal femur osteotomy. Examination of the intra-articular knee joint revealed that the tibial articular surface was irregular and had severe posterior angulation. Next, we performed an osteotomy of the tibia. A Kirschner wire was inserted perpendicular to the tibial bone axis in the sagittal plane from the anterior tibia toward the posterior hinge point as determined preoperatively, and another Kirschner wire was inserted from 10 mm proximal to the first insertion point toward the hinge point. With these osteotomy lines as references, we performed a wedge osteotomy using a flat osteotome and removed the bone fragments. We then performed an oblique osteotomy of the fibula, manually corrected the alignment, and temporarily fixed the bone using a Kirschner wire. A four-surface osteotomy of the femur was subsequently performed using the epicondylar axis as a reference, followed by an osteotomy of the tibia using an intramedullary guide with the lowest point of the tibial articular surface as a reference. The osteotomy did not affect the patellar tendon enthesis. Finally, we detached the superficial layer of the medial collateral ligament enthesis and achieved good medial and lateral ligament balance. We utilized a long-stem implant on the tibia and a resurfacing implant on the femur. To reinforce rotational stability of the corrective osteotomy site, we used a one-third tubular plates. For postoperative therapy, the patient began range-of-motion exercises the day after the operation and began partial weight bearing at 3 weeks postoperatively. We permitted full weight bearing at 6 weeks postoperatively, and the patient was able to walk without assistance. After 17 months, he was diagnosed with late infection of the left knee, which improved with irrigation, debridement, extraction of a one-third tubular plates and antibiotic treatment. At 2-year follow-up, the patient had no pain while walking and range of motion was 0° extension to 125° flexion. There was no evidence of recurrence of infection. Radiographs at 24 months postoperatively showed that bone union of the osteotomy site was achieved (Fig. 3).

## Conclusion

Tibial plateau fractures are often complicated by damage to the joint cartilage, meniscus, and ligaments, leading to OA [3]. In the present case, knee OA was confirmed 21 years after the injury and nonunion was observed in the posterior tibia. There are two TKA methods available to correct knee OA accompanying coronal plane deformity: an intra-articular method and an extra-articular method. If the deformity is severe, using an intra-articular correction method increases the number of osteotomies and induces soft tissue imbalance and instability. Moreover, since thorough coverage at the time of implant placement is difficult to obtain, corrective osteotomy is recommended. The criteria for corrective osteotomy have mostly been reported in coronal plane deformities, with varying degrees of tibial deformities suitable for the procedure; Wang et al. used tibial deformity greater than 30°, while Radke et al. used deformity greater than 15° [1, 2]. Meehan et al. reported a case in which they achieved good clinical outcomes with a 35° tibial valgus corrective osteotomy performed concurrently with TKA for knee OA associated with malunion of a tibial plateau fracture [4]. However, using sagittal plane corrective osteotomy for a sagittal plane deformity is uncommon, and the number of case reports is low. Posterior angulation in the sagittal plane is often increased after tibial plateau fractures [5]. For placement of the artificial joint at the tibial articular surface, if the osteotomy line uses the lowest point of the articular surface as a reference point, the osteotomy affects the tibial tuberosity. If it is proximal to the lowest point of the tibial articular surface, coverage of the articular surface by the implant is significantly decreased. For these reasons, correcting the alignment of the sagittal plane similar to the coronal plane is important. In the current case, although the coronal plane varus deformity was 9°, a high degree (27°) of sagittal plane flexion deformity was present. We therefore applied sagittal plane corrective osteotomy. There are two methods for performing corrective osteotomy with TKA: one-stage surgery using a long-stem TKA and two-stage surgery where corrective osteotomy is performed first, followed by performing TKA after bone union is achieved. Some issues are seen with one-stage surgery compared to two-stage, such as a more complicated and invasive procedure with difficulties in correcting alignment. However, the many advantages of one-stage surgery include preventing infection and arthrogryposis caused by multiple surgeries, relieving the pain of knee OA after one surgery, and returning to a normal routine earlier with shorter treatment and recovery times [6–8]. Two-stage surgery, particularly in elderly patients, lowers joint function due to longer treatment periods. Therefore, we performed one-stage surgery. The patient began range-of-motion exercises the day after the operation and was able to walk without assistance by 6 weeks postoperatively. Plain radiographs taken 24 months after the operation verified that bone union was achieved. We believe this surgical method is beneficial, not only for enabling correction with good alignment but also because it achieves reinforced early fixation and facilitates patients to start walking at an early stage of recovery.

## Consent

Written informed consent was obtained from the patient for publication of this Case Report and any accompanying images.

## Abbreviations

TKA: total knee arthroplasty
OA: osteoarthritis
3D: three-dimensional

## References

[1] Wang JW, Wang CJ (2002). Total knee arthroplasty for arthritis of the knee with extra-articular deformity. J Bone Joint Surg Am.

[2] Radke S, Radke J (2002). Total knee arthroplasty in combination with a one-stage tibial osteotomy: a technique for correction of a gonarthrosis with a severe (>15 degrees) tibial extra-articular deformity. J Arthroplast.

[3] Honkonen SE (1995). Degenerative arthritis after tibial plateau fractures. J Orthop Trauma.

[4] Meehan JP, Khadder MA, Jamali AA, Trauner KB (2009). Closing wedge retrotubercular tibial osteotomy and TKA for posttraumatic osteoarthritis with angular deformity. Orthopedics.

[5] Streubel PN, Glasgow D, Wong A, Barei DP, Ricci WM, Gardner MJ (2011). Sagittal plane deformity in bicondylar tibial plateau fractures. J Orthop Trauma.

[6] Moyad TF, Estok D (2009). Simultaneous femoral and tibial osteotomies during total knee arthroplasty for severe extra-articular deformity. J knee Surg.

[7] Xiao-Gang Z, Shahzad K, Li C (2012). One-stage total knee arthroplasty for patients with osteoarthritis of the knee and extra-articular deformity. Int Orthop.

[8] Papagelopoulos PJ, Karachalios T, Themistocleous GS, Papadopoulos E, Savvidou OD, Rand JA (2007). Total knee arthroplasty in patients with pre-existing fracture deformity. Orthopedics.

